# Sonographic findings in mid-aortic syndrome

**DOI:** 10.1259/bjrcr.20200123

**Published:** 2020-10-29

**Authors:** Kieran Kusel, Hannah Zubrowski, Yuranga Weerakkody

**Affiliations:** 1 Department of Radiology, Royal Perth Hospital, Perth, WA, Australia

## Abstract

Mid-aortic syndrome (MAS) is an uncommon condition characterised by narrowing of the distal descending thoracic or abdominal aorta. While CT, MR and conventional angiography findings in MAS are well described, there have been very few cases which clearly document the sonographic features of this condition. This case report demonstrates the utility of ultrasound in the investigation of MAS and summarises the current literature surrounding the condition.

## Introduction

Mid-aortic syndrome (MAS) is an uncommon condition characterised by narrowing of the distal descending thoracic or abdominal aorta. It most commonly affects children and young adults who typically present with severe arterial hypertension and non-specific symptoms such as headache, dyspnoea and fatigue. If left untreated, MAS can result in end-organ dysfunction secondary to hypertension, including renal failure, encephalopathy, retinopathy, coronary artery disease, congestive heart failure and cerebrovascular accidents. While imaging findings in CT, MR and conventional angiography are well described, sonographic findings in MAS such as aortic luminal narrowing and corresponding changes to blood flow dynamics are not well documented. Understanding the sonographic features of MAS is particularly important because ultrasound is often used as a first-line investigation when assessing for secondary causes for hypertension in these patients.

## Clinical presentation

A 43-year-old male was referred to our service for a renal artery Doppler ultrasound as part of investigation of resistant hypertension. He had a systolic blood pressure of up to 260 mm Hg despite triple antihypertensives. He had a history of recurrent abdominal pain requiring multiple previous hospital admissions but no history of headache, nausea or lower limb claudication. He had normal renal function.

## Imaging findings

**Figure 1.**
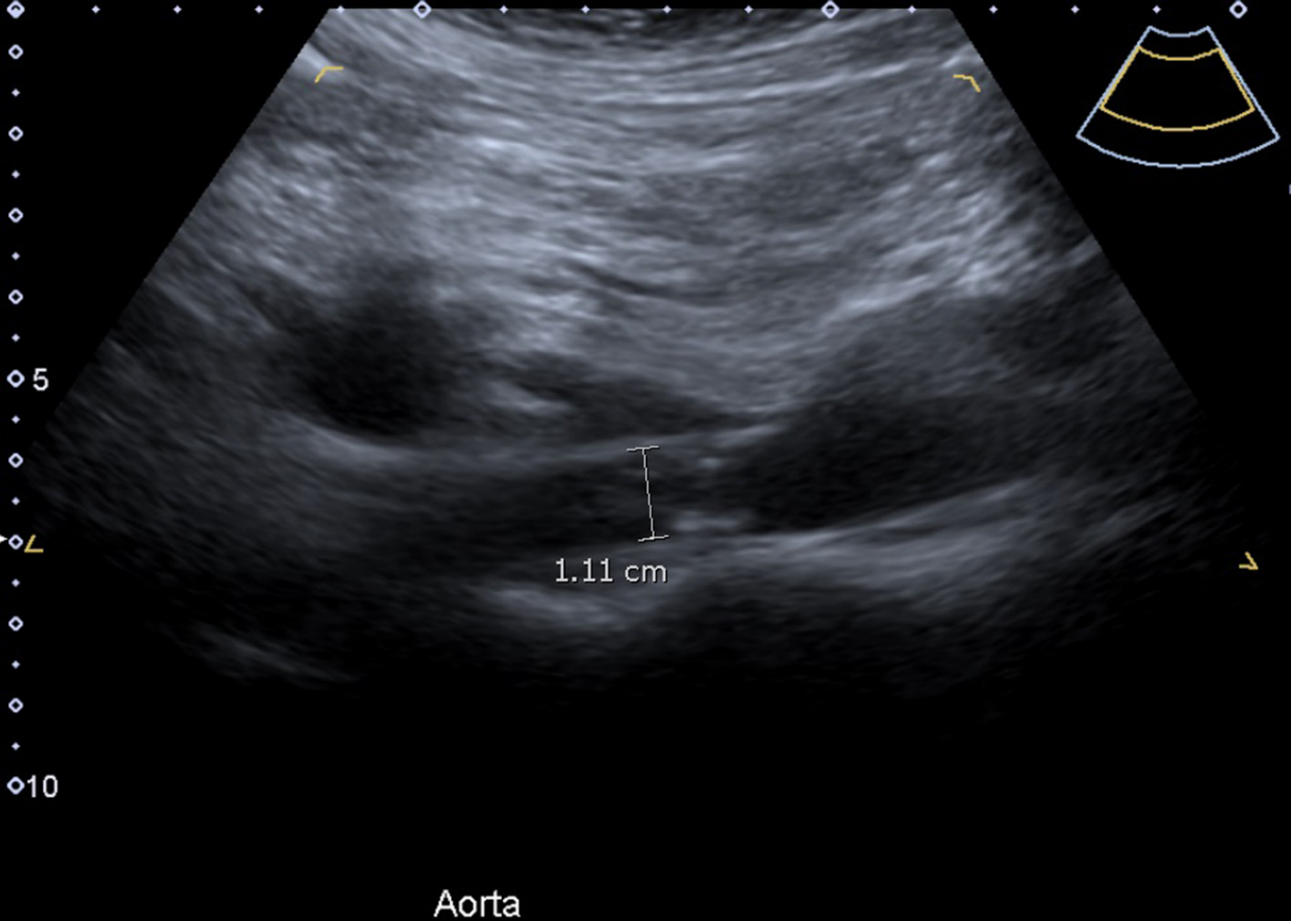
B-mode ultrasound image of the suprarenal abdominal aorta in longitudinal orientation demonstrates irregular narrowing of the aorta with a diameter of 11 mm (where a normal diameter is 21–25 mm).

**Figure 2.**
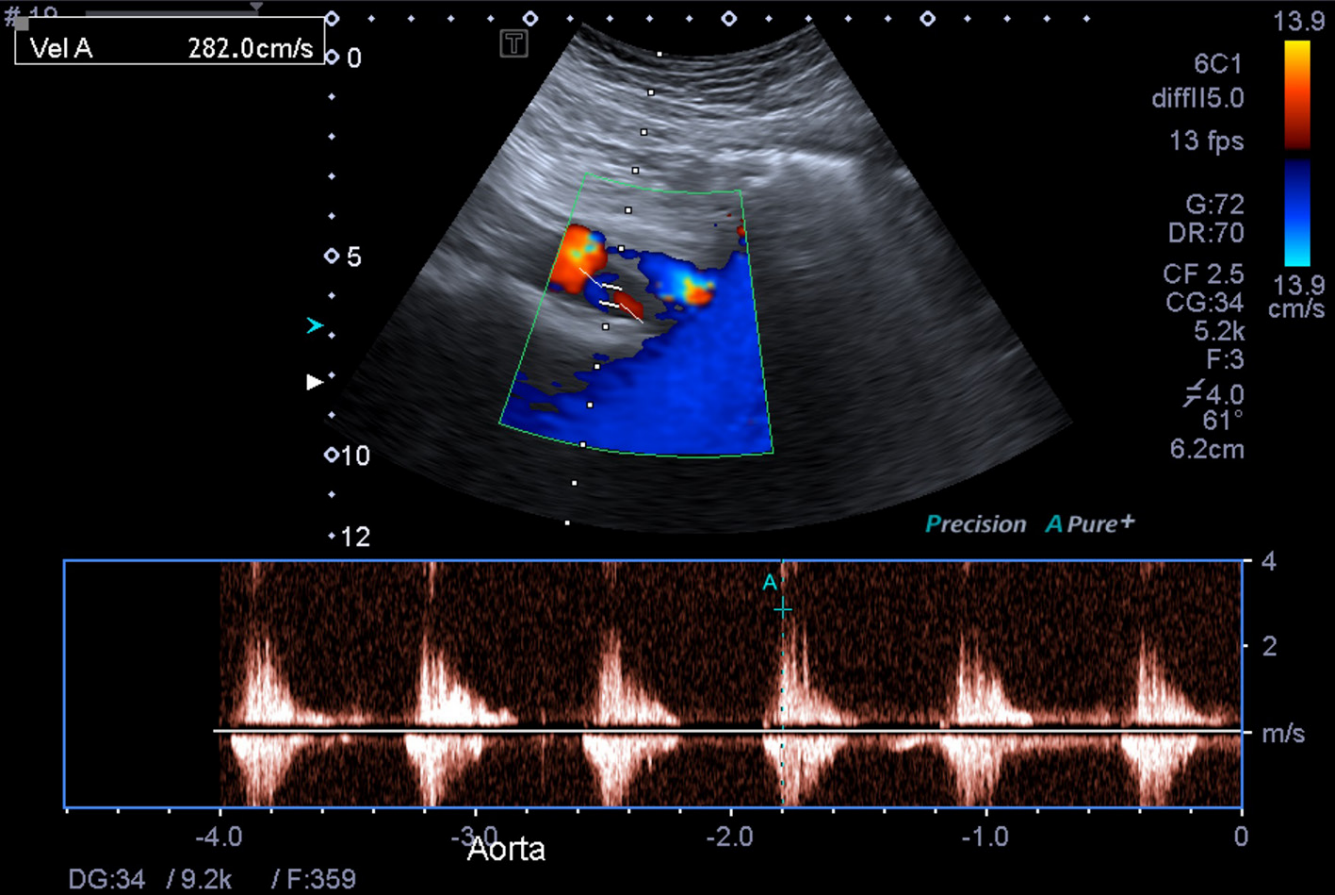
Colour and pulse wave Doppler ultrasound demonstrates turbulent flow and an elevated peak systolic velocity within the narrowed suprarenal abdominal aorta.

**Figure 3.**
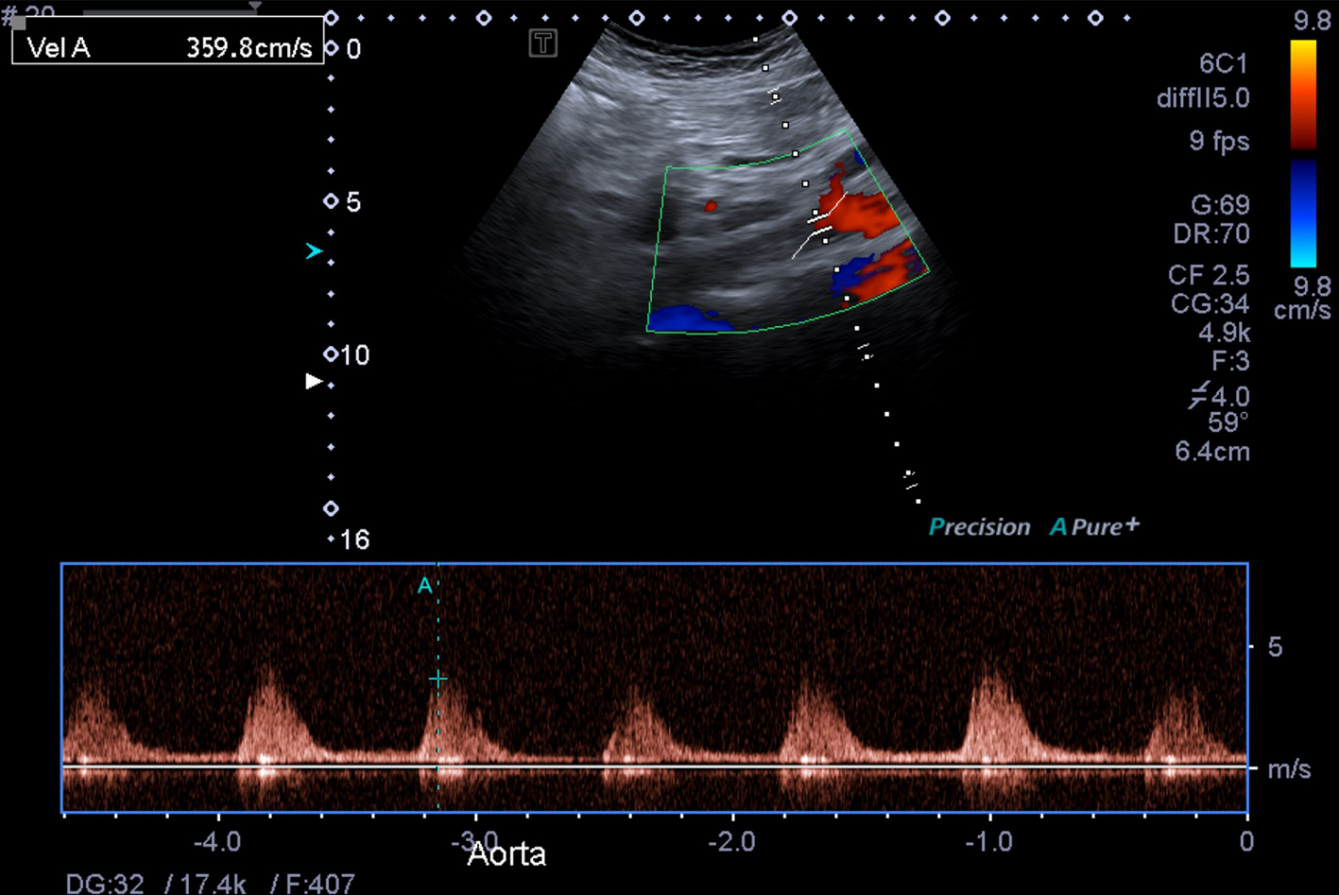
Pulse wave Doppler ultrasound demonstrates an elevated peak systolic velocity within the narrowed suprarenal abdominal aorta of 360 cm/s (where a normal velocity is up to 150 cm/s).

Grey-scale ultrasound imaging demonstrated narrowing of the suprarenal abdominal aorta with a diameter of approximately 11 mm (Figure 1, a normal diameter at the level of the coeliac trunk is 21–25 mm).^1^ There was associated turbulent flow and elevated peak systolic velocities measuring up to 360 cm/sec in the narrowed segment (Figures 2 and 3, normal peak systolic velocity is considered to be up to 150 cm/s.^2^ Distally, the abdominal aorta diameter increased and an accompanying reduction in peak systolic velocities was seen.

**Figure 4.**
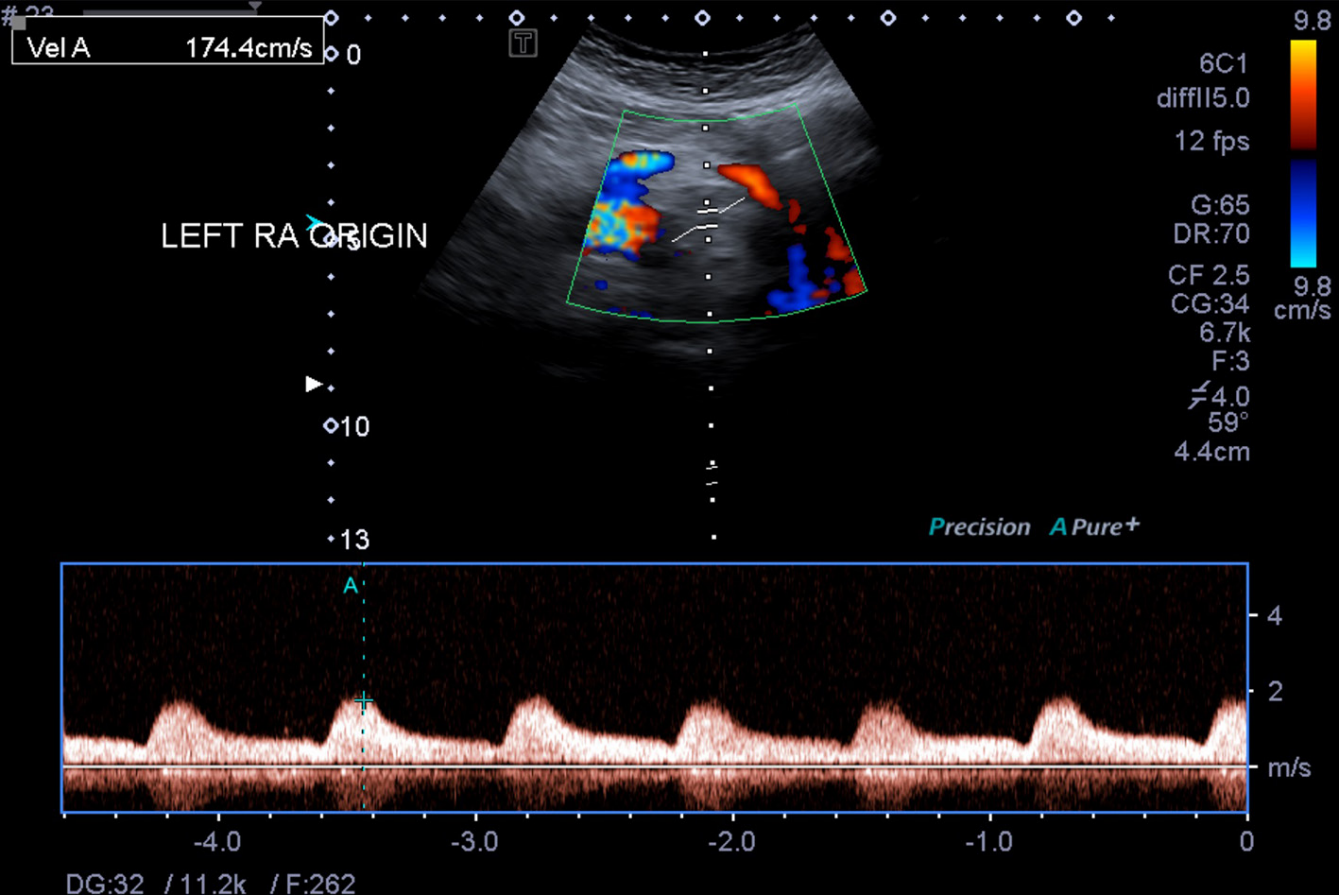
Pulse wave Doppler ultrasound demonstrates a relative reduction in abdominal aorta peak systolic velocity with a tardus parvus waveform at the level of the left renal artery ostium.

A tardus parvus waveform was demonstrated in the abdominal aorta at the level of the left renal artery (Figure 4). The main renal arteries were patent with normal flow velocities of 89–139 cm/s and 95–126 cm/s on the right and left, respectively. Both renal veins were patent with normal flow.

The right and left kidneys demonstrated normal sonographic appearances and size. Normal resistive indices with a mean of 0.67, acceleration times (all <0.07 sec) and acceleration indices (all >350 cm/sec^2^) were recorded in each kidney.

**Figure 5.**
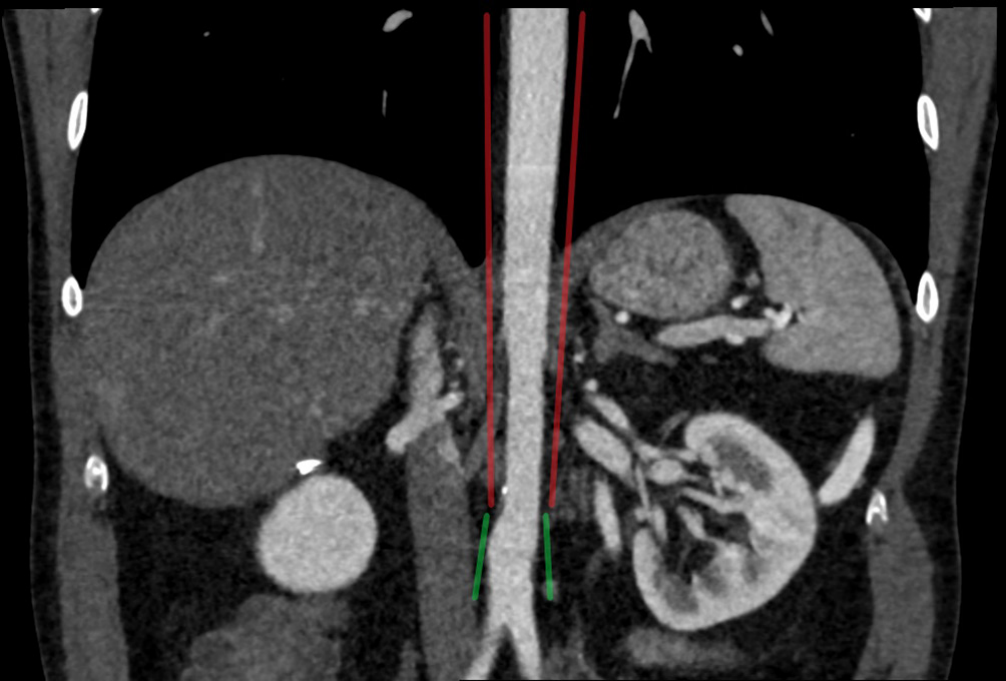
Coronal CT angiogram of the distal descending thoracic aorta and abdominal aorta demonstrates progressive narrowing (indicated in red) and an increase in diameter distally (indicated in green).

**Figure 6.**
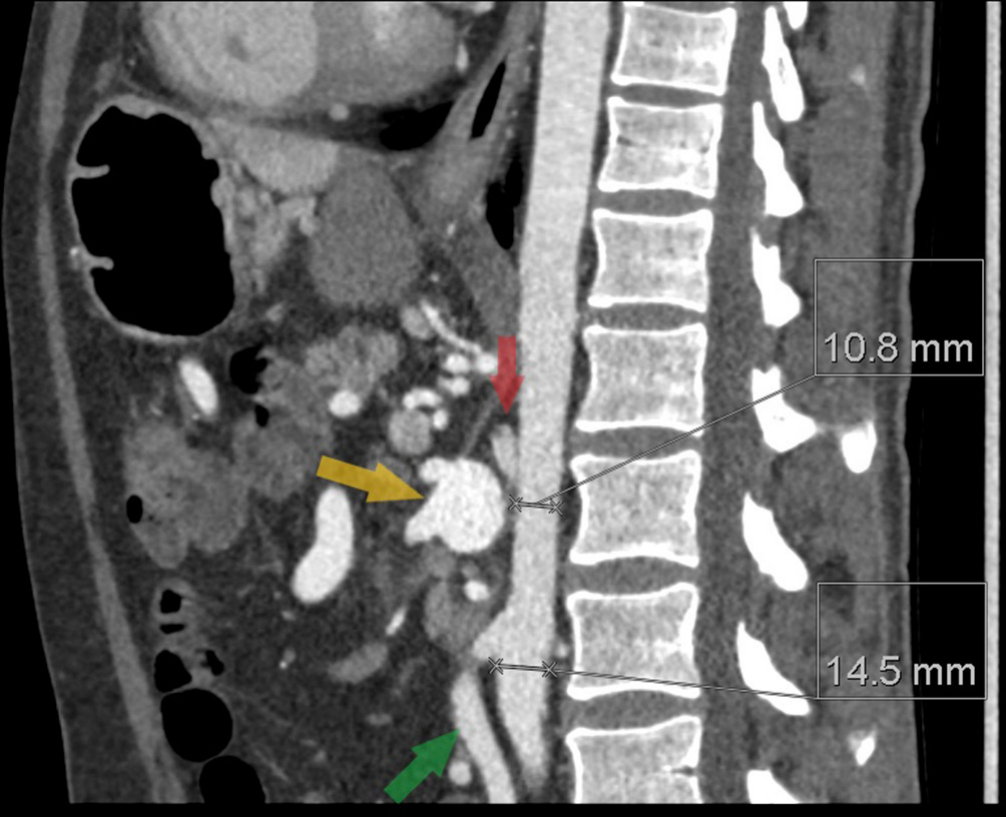
Sagittal CT angiogram demonstrates progressive narrowing of the distal descending thoracic and abdominal aorta to the level of the renal arteries (10.8 mm) distal to which it increases in calibre (14.5 mm). There is atresia of the proximal coeliac axis (indicated by the red arrow) and superior mesenteric artery (the origin not visible in this image), and an enlarged inferior mesenteric artery (indicated by the green arrow). Coeliac axis and SMA branches originate from a 22 mm aneurysmal dilatation of the proximal SMA (indicated by the yellow arrow) where there is an anastomosis with the IMA via a dilated marginal artery of Drummond.

Review of a previous CT angiogram of the abdomen demonstrated progressive narrowing of the distal descending thoracic and abdominal aorta. Aortic diameter measured 10.8 mm at the ostia of the renal arteries. Inferiorly, the diameter of the aorta increased to 15 mm at the origin of the inferior mesenteric artery (where a normal diameter of the aorta is usually 18–22 mm^1^ (Figures 5 and 6)). Notably, there was atresia of the origin of the coeliac axis and superior mesenteric artery. An enlarged inferior mesenteric artery and marginal artery of Drummond anastomosed with the proximal superior mesenteric artery where there was a 22 mm aneurysm. Coeliac axis and superior mesenteric artery branches arose from this point.

## Discussion

Mid-aortic or middle aortic syndrome (MAS) is an uncommon condition characterised by narrowing of the distal descending thoracic or abdominal aorta.^3–5^ Narrowing may be segmental or diffuse and involve the renal arteries (in 63–90% of cases) and visceral branches (in 25–50% of cases the coeliac axis and superior mesenteric artery are involved, with the inferior mesenteric artery almost never affected).^3–7^ MAS is usually diagnosed in childhood or adolescence, however, cases are occasionally detected in adults.^8–10^ Patients with MAS typically present with severe arterial hypertension (90% of patients) which is commonly unresponsive to pharmacological antihypertensive treatment.^3,4,11^ Although many patients are asymptomatic, symptoms can include headache, dyspnoea, early fatigue on exertion or epistaxis. Patients may also present with complications from hypertension such as encephalopathy, retinopathy, coronary artery disease, congestive heart failure or cerebrovascular accidents.^3,9^ Leg claudication and intestinal ischaemia are infrequent, thought largely to be due to the gradual development of stenosis allowing effective collateral circulation pathways to form.^4,11^ A systematic review of 630 children with MAS by Rumman *et al*. in 2015 found that abdominal angina was present in only 4.1% of cases.^3^ Along with hypertension, examination findings may include weak or absent femoral pulses, a difference in blood pressure between upper and lower limbs, an ejection systolic murmur, and an abdominal bruit.^3–5^ Associated renal impairment is common.^4^

The aetiology of MAS is unknown and, although a number of associations with genetic and acquired diseases have been proposed, the majority of cases are idiopathic.^3,4^ Congenital failure of normal fusion of the two dorsal aortas during the fourth week of gestation has been proposed, and links with genetic diseases such as neurofibromatosis and Williams syndrome or acquired inflammatory diseases such as Takayasu’s arteritis or intrauterine rubella have also been considered.^3,4^

While intra-arterial angiography of the aorta was previously the gold standard diagnostic test for MAS, CT angiography and MR angiography are now more widely used for evaluation and can define the extent of disease and guide surgical or endovascular management.^4,12^ Radiation exposure from CT and safety issues of gadolinium in MRI are, however, important considerations particularly because MAS most commonly affects children and young adults, many of whom can have impaired renal function.^4^

Surprisingly, there have been very few case reports documenting the sonographic features of MAS, particularly given that abdominal ultrasound is frequently used as a first-line investigation assessing for secondary causes of hypertension in these patients. Furthermore, lack of exposure to ionising radiation, low cost and widespread availability make it a useful modality to aid in diagnosis.^4^ Documented sonographic features of MAS include segmental narrowing or tortuosity of the aorta, aliasing or turbulent flow on colour Doppler, and elevated peak systolic velocities in the narrowed portion with tardus parvus waveforms in vessels distal to the stenosis on pulse wave Doppler.^4,6,13^ In the case discussed above, the first sonographic finding was a tardus parvus waveform at the origin of the left renal artery, suggestive of an upstream stenosis. The abdominal aorta was then imaged and demonstrated irregular narrowing of the suprarenal portion with elevated peak systolic velocities of 233 cm/s and 360 cm/s in its proximal and middle portions with subsequent reduction in velocity to 174 cm/s distally. We demonstrate that ultrasound is a useful diagnostic tool since it not only demonstrates luminal narrowing, but can also assess the functional effect of the stenosis.

Management of MAS is aimed at controlling blood pressure and preserving end-organ function. Treatment usually involves pharmacological antihypertensive therapy for those with mild-to-moderate stenosis. Often, however, endovascular or surgical treatment is required in patients with refractory hypertension and diminished renal function.^3,12–15^

## Conclusion

Mid-aortic syndrome has traditionally been investigated with CT, MR and conventional angiography and imaging findings have been well-defined. However, MAS most commonly affects young patients who typically present with severe arterial hypertension and in whom ultrasound is often the first imaging modality requested. Despite this, there are very few reported cases which clearly document the sonographic findings in this condition. Ultrasound is a useful diagnostic tool that can demonstrate luminal narrowing of the aorta and functional effects of the stenosis. It also has the benefits of a lack of exposure to ionising radiation and does not have the safety concerns surrounding gadolinium use in MRI. This is particularly important in children and young adults with impaired renal function – those who are most likely to be diagnosed with this condition.

## Learning points

1. Mid-aortic syndrome is an uncommon condition characterised by narrowing of the distal descending thoracic or abdominal aorta.
2. CT, MR and conventional angiography have traditionally been used in the investigation of this disease.
3. Ultrasound is an effective imaging modality which can demonstrate luminal narrowing and altered flow parameters. It is often the first-line imaging investigation requested. Understanding the sonographic features will help to improve detection of this uncommon disease.
